# Safety and Effectiveness of Subcutaneous Immunotherapy with an Undiluted Glutaraldehyde-Polymerized Pollen Extract Mixture in Adults and Children with Allergic Rhinitis with or Without Asthma Due to Olive and Grass Pollen

**DOI:** 10.3390/diseases14090321

**Published:** 2026-09-04

**Authors:** Paula López-González, María Antonia Padial Vilchez, María Teresa Palomeque Rodríguez, María Galicia Dávila-Fernández, Virginia Bellido Linares, Emilio Funes Vera, Conchita Cordobés Durán, Ana Montoro Ferrer, Estefanía Moreno Mata, Victoria Villalobos Violán, Laura Ortega-Martín, Aída Gómez-Cardenosa

**Affiliations:** 1 Allergy Department, Infanta Leonor University Hospital, 28031 Madrid, Spain; paula.lopez@salud.madrid.org; 2 Allergy Department, Infanta Sofía University Hospital, 28702 Madrid, Spain; 3 Allergy Department, Nuestra Señora del Perpetuo Socorro University Hospital, 02006 Albacete, Spain; maytepalomeque03@hotmail.com; 4 Allergy Department, La Cruz Roja San José y Santa Adela Central University Hospital, 28003 Madrid, Spain; galicia.davila@salud.madrid.org; 5 Allergy Department, El Tomillar Hospital, 41500 Sevilla, Spain; virlinb@hotmail.com; 6 Allergology Unit, Hospital Universitario Rafael Méndez, 30817 Murcia, Spain; efunesvera@gmail.com; 7 Allergy Department, Badajoz University Hospital, 06006 Badajoz, Spain; cordobesc@hotmail.com; 8 Allergy Department, Villarrobledo University Hospital, 02600 Albacete, Spain; a.montoroferrer@gmail.com; 9 Allergy Department, Mancha Centro University Hospital, 13600 Ciudad Real, Spain; esmormat@hotmail.com; 10 Allergy Department, Fuenlabrada University Hospital, 28942 Madrid, Spain; victoria.vilalobos@salud.madrid.org; 11 Medical Affairs Department, Diater Laboratories, 28919 Madrid, Spain; l.ortega@diater.com

**Keywords:** allergen mixtures, allergic asthma, allergic rhinitis, glutaraldehyde-polymerized allergoids, grass pollen, *Olea europaea*, polysensitization, subcutaneous allergen immunotherapy

## Abstract

Background/Objectives: To evaluate the tolerability and effectiveness of subcutaneous immunotherapy (SCIT) with a glutaraldehyde-polymerized undiluted mixture of grass and olive pollen in adults and children with allergic respiratory disease in routine clinical practice. Methods: Observational, ambispective, controlled multicenter study including patients ≥ 5 years with allergic rhinitis/rhinoconjunctivitis +/− asthma due to olive and grass pollen. Patients initiating SCIT with *Olea europaea*/grass pollens undiluted mixtures were included in the O&G group, and those continuing symptomatic treatment in the untreated (UT) group. Safety (primary objective) was assessed as the incidence of adverse reactions. Effectiveness variables (symptoms, control, and medication use) were compared during the pollen season before and after AIT; patients and investigators reported perceived satisfaction. Results: We included 218 patients in the O&G group (children, 23.4%; adolescents, 13.8%; adults, 62.8%) with a mean (SD) age of 26.5 (15.6) years, and 94 in the UT group. At the time of evaluation, patients had received treatment for a mean (SD) of 10.6 (2.5) months. Seventeen patients (7.8%) experienced 18 adverse reactions in total, all local (12 in adults, 6 in children; mean overall rate: 0.8%). Rhinitis frequency shifted from predominantly persistent to intermittent (−64.6% in persistent) (*p* < 0.0001), with significantly improved intensity and control overall and across age groups in the O&G group but not in the UT group. Patients with asthma symptoms decreased by −46.6% (*p* < 0.0001), along with improved asthma classification, treatment steps, and control overall (O&G group), and across most age groups. Changes in conjunctivitis symptoms followed a similar trend. Symptomatic medication use significantly decreased overall (O&G group) and across specific age groups. Patients and investigators perceived decreased symptoms and medication use after AIT. Conclusions: SCIT with a glutaraldehyde-polymerized undiluted allergen mixture of olive/grass pollen extract demonstrated safety and effectiveness to treat allergic rhinitis and asthma in adults and children in a real-world setting.

## 1. Introduction

Allergic respiratory disease (ARD), including allergic rhinitis and asthma, comprises inflammatory conditions of the airways and represents one of the most common chronic conditions worldwide [1,2]. Despite variations due to geographical differences, disease definitions, and methodologies used, the worldwide prevalence of allergic rhinitis has been estimated at a median 18.1% in adults and ranges from 10.48% to 18.12% in children [3,4]. Allergic rhinitis often progresses to allergic asthma, and both conditions frequently coexist [1]. Asthma worldwide prevalence has been estimated to range between 5.3 and 11.5% [5]. ARD significantly impairs patients’ quality of life, as well as school and work performance and daily activities, representing a substantial burden for patients and healthcare systems, with additional indirect socioeconomic costs [6,7].

Among the different allergens causing ARD, seasonal exposure to pollen grains from different plant species leads to sensitization and triggers clinical symptoms [8]. The prevalence of sensitization to different pollens is geographically variable according to exposure patterns depending on species distribution and pollination periods [8]. Moreover, while in Northern Europe, the pollination periods of the most allergenic species are separated, in Southern Europe, successive and overlapping pollen seasons result in complex and prolonged exposures, frequently leading to poly-sensitization [8]. In Mediterranean areas, such as Spain, due to overlapping pollen seasons and high environmental exposure, co-sensitization to olive and grass pollens is common, with estimated prevalences ranging between 50% and 76% among patients with ARD, often translating into clinically relevant responses to multiple allergens (i.e., polyallergy) [9,10,11].

Allergen immunotherapy (AIT), which consists of the sublingual (SLIT) or subcutaneous (SCIT) administration of allergen extracts at controlled doses, targets the underlying cause of the disease and has been shown to alleviate symptoms, improve disease control, and modify the disease course, providing a more sustained effect than conventional pharmacotherapy [12]. While initial approaches were based on the administration of native extracts, multiple AIT products containing chemically modified allergen extracts, known as allergoids, have been developed to improve their safety and efficacy [13]. Among those, allergens polymerized with glutaraldehyde are widely used in SCIT products and have been associated with improved safety compared to native extracts, including a lower incidence of systemic reactions, allowing the administration of higher allergen doses while maintaining tolerability [13]. This is particularly relevant in patients requiring treatment with multiple clinically relevant allergens, as undiluted mixtures avoid the dilution of individual components and maintain each allergen extract at a 100% concentration.

In polyallergic patients, AIT strategies need to target more than one allergen to achieve an optimal clinical benefit [14]. In this setting, administration of allergen mixtures, such as combinations of olive and grass pollen extracts, represents a practical approach in routine clinical practice [14]. However, the use of mixtures raises specific considerations, since the efficacy of AIT is dose-dependent and adequate allergen doses should be maintained for each component [15,16]. At the same time, AIT safety is also dose-dependent and therefore, increasing allergen exposure in AIT mixtures may impact tolerability, requiring an optimal benefit–risk balance [17,18]. Despite limited guidance in European recommendations regarding the use of mixed products, their use remains common in real-world settings to address the needs of polyallergic patients [14].

Evidence regarding the safety and effectiveness of AIT with undiluted mixtures is still scarce. In addition, evidence specifically evaluating undiluted glutaraldehyde-polymerized mixtures of olive and grass pollen extracts under real-world conditions remains limited. This is particularly relevant in Mediterranean areas where co-sensitization to both allergens is common and maintaining undiluted allergen extract mixtures represents a practical challenge in routine clinical practice.

This observational, ambispective study aimed to evaluate the tolerability of SCIT with an undiluted mixture of wild grasses and *Olea europaea* pollen extract polymerized with glutaraldehyde in patients with rhinitis, with or without asthma, in routine clinical practice. Secondary objectives were to assess the effectiveness of the AIT and the relationships between tolerability and demographic, clinical, and treatment variables.

## 2. Materials and Methods

### 2.1. Study Design and Population

This was an ambispective (retrospective and cross-sectional), controlled, real-world multicenter study including patients over four years old with allergic rhinitis, with or without controlled asthma, due to sensitization to house dust mites (*Dermatophagoides pteronyssinus* and *Dermatophagoides farinae*, or *Dermatophagoides pteronyssinus* and *B. tropicalis*) or pollens from *O. europaea* and wild grasses. Patients were diagnosed according to skin prick tests (SPT) and/or specific IgE (sIgE) determinations and subsequently started AIT at 30 participating centers according to routine clinical practice between 1 September 2021 and 1 June 2022.

This report is a subanalysis of the cohort who started AIT with undiluted mixtures of *O. europaea* + wild grasses pollen extracts. The subanalysis in patients receiving mixtures of *D. pteronyssinus* and *D. farinae* extracts polymerized with glutaraldehyde have been published elsewhere [19]. All patients who started the AIT were included in the treatment group (O&G), regardless of the total number of doses received, whereas patients sensitized to the study allergens (*D. pteronyssinus* and *D. farinae*, *O. europaea* and wild grasses, or *D. pteronyssinus* and *B. tropicalis*) who did not start the AIT during the same period and remained on conventional pharmacological treatment to control their symptoms were included in the control group (untreated, UT). Patients whose medical history was unavailable or who had received AIT with *O. europaea* and/or wild grasses pollen extracts within the previous five years were excluded from the study. Data were collected from medical records between October 2022 and March 2023 and included data from before and after starting the AIT, the latter corresponding to the annual follow-up visit according to routine clinical practice. Moreover, questionnaires to evaluate the self-perceived effectiveness of the AIT, including symptoms, overall use of medication, and satisfaction with treatment, were administered to patients and investigators during the recruitment visit and after patients’ inclusion, respectively (cross-sectional data).

The study was conducted according to the principles of the Helsinki Declaration and later amendments and the local data protection regulations (Spanish Organic Law 15/1999, of 13 December). All included patients and legal representatives of patients < 18 years old signed a written informed consent before any information was collected. The Independent Ethics Committee (Comité de Ética para la Investigación con medicamentos, CEIm) of Getafe University Hospital (Madrid, Spain) approved the study protocol.

This manuscript was prepared and revised in accordance with the Strengthening the Reporting of Observational Studies in Epidemiology (STROBE) statement [20].

### 2.2. Study Medication

The AIT assessed in this study contained an undiluted mixture of *O. europaea* and wild grasses pollen extracts (*O. europaea* 100%/wild grasses 100%) polymerized with glutaraldehyde in an aqueous suspension for subcutaneous administration (Polymerized 100, Diater Laboratories, Madrid, Spain). The wild grass pollen component consisted of a mixture of *Lolium perenne*, *Poa pratensis*, *Phleum pratense*, and *Dactylis glomerata*, which are among the most clinically relevant grass species associated with seasonal allergic respiratory disease in Mediterranean areas.

It was administered in two phases: an up-dosing phase consisting of the administration of increasingly higher doses of the extract mixture, followed by a maintenance phase, consisting of monthly injections of the 0.5 mL maintenance dose for three years. In the build-up phase, AIT administration could follow two possible schedules: a cluster schedule consisting of a 0.2 mL and a 0.3 mL dose on the same day at a 30 min interval and a conventional two-week schedule consisting of the administration of a single dose of 0.2 mL at day 1 and 0.5 mL at day 8.

### 2.3. Objectives

The primary objective was to evaluate tolerability, assessed as the incidence of adverse reactions (ARs). Secondary tolerability objectives included the evaluation of ARs according to the administration phase, clinical manifestations of allergy (rhinitis vs. rhinoconjunctivitis and asthma vs. no asthma), and demographic variables, namely age and sex. The effectiveness of AIT was evaluated as a secondary objective based on changes in allergic rhinitis and allergic asthma symptoms (presence, frequency, intensity/severity, and control) and medication use from baseline to the routine follow-up visit after AIT initiation. The effectiveness evaluation for both allergic rhinitis and allergic asthma was based on the comparison between baseline and the annual follow-up visit performed approximately 12 months after AIT initiation according to routine clinical practice. These variables were evaluated in the O&G and UT groups. AIT effectiveness was also evaluated based on patient- and investigator-reported changes in symptoms and medication intake and satisfaction with treatment.

### 2.4. Variables and Assessments

Variables included in the evaluation of the primary objective were incidence of systemic reactions (SRs) and local reactions (LRs) per injection administered (ARs/100 injections or percentage of injections with ARs). In addition to type, we classified reactions as immediate or late according to a time of onset within 30 min after injection or >30 min after injections, respectively. SRs were classified according to the World Allergy Organization (WAO) criteria [21]. Tolerability evaluation included maintenance doses administered during the pollen season.

To evaluate AIT effectiveness, allergic rhinitis was classified according to the Allergic Rhinitis and its Impact on Asthma (ARIA) criteria, (Table 1) [22]. Intermittent allergic rhinitis was defined as symptoms occurring on fewer than 4 days per week or for fewer than 4 consecutive weeks, whereas persistent allergic rhinitis was defined as symptoms occurring on 4 or more days per week and for 4 or more consecutive weeks. Asthma was classified according to the Guía Española para el Manejo del Asma 5.0 (GEMA), the Spanish version of the Global Initiative for Asthma (GINA) guidelines [23].

GINA uses different classifications in the adult and pediatric population, the latter being more detailed and focused on the evolution of asthma control. The symptomatic medications considered included oral, nasal, and ocular antihistamines, oral, nasal, and inhaled corticosteroids, β2 inhaled agonists, and anti-leukotrienes, and their use was evaluated as yes and no. Effectiveness was compared between the O&G groups and the UT group and, additionally in the O&G group, changes were evaluated by age, including children (5 to 11 years), adolescents (12 to 17 years), and (adults ≥ 18 years).

Patients and investigators reported the self-perceived effectiveness of the AIT compared to before starting the AIT (less, same, and more) using specific ad hoc questionnaires, encompassing rhinitis, conjunctivitis, and asthma symptoms, and the overall use of medications. Moreover, patients and investigators rated their global satisfaction with the treatment on a visual analog scale (VAS) ranging from 0 to 100, where 100 represented the highest satisfaction level.

Additional variables considered were demographic characteristics, and characteristics of the allergic disease at baseline, including diagnoses and sensitizations, assessed using skin prick tests (SPT) to *O. europaea* and wild grasses, and quantification of specific IgE (sIgE) to the major allergens Ole e 1, Phl p 1, and Phl p 5, with variations among hospitals depending on clinical routine assessments (positive sensitization sIgE ≥ 0.35 KU/L). AIT treatment characteristics included time from AIT treatment initiation to data collection (months) and AIT schedule.

### 2.5. Statistical Analysis and Sample Size

Estimation of the sample size was based on a 20% prevalence of AR in the Spanish population [24]. A total of 502 patients were deemed necessary to evaluate the primary objective (safety) with a 3.5% precision and a 95% confidence interval (CI) without the need to replace patients.

Categorical variables were presented as frequencies and percentages, and quantitative variables with the mean and standard deviation or the median and interquartile range (IQR, 25th and 75th percentiles). Relationships between the incidence of adverse reactions and patient characteristics, including age, sex, rhinitis diagnosis, and asthma diagnosis were evaluated using the Chi square test with a 95% confidence level. Variables before and after AIT were compared using Fisher’s one-sided statistic, the McNemar test, and the Chi square test with a 95% confidence level. Statistical significance was set at a *p* < 0.05. All statistical analyses were performed using the statistical package SAS system v9.4 and subsequent versions.

## 3. Results

### 3.1. Demographic, Clinical and Treatment Characteristics

Of 465 patients recruited, eight were not valid for analysis due to missing variables. Of the remaining 457 valid patients, 94 did not receive AIT and were included in the UT group, and 363 received AIT: 218 (47.7%) with mixtures of *Olea europaea*/wild grasses extracts (O&G group), 130 (28.45%) with mixtures of *D. pteronyssinus*/*D. farinae* extracts, and 15 (3.28%) with mixtures of *D. pteronyssinus*/*Blomia tropicalis* extracts.

In the O&G group (n = 218), mean (SD) age was 26.5 (15.6) years, and 51.4% of patients were male (Table 2). Approximately two-thirds of patients were adults (62.8%) and 37.2% were pediatric patients; 23.4% were children (5–11 years) and 13.8% were adolescents (12–17 years). Sex and age distribution was similar in the UT group. Regarding diagnoses, in addition to rhinitis, almost all patients had conjunctivitis (91.7%) and approximately two-thirds (63.3%) were diagnosed with concomitant asthma. The most frequent diagnoses combinations were rhinitis, conjunctivitis, and asthma (59.2%) and rhinitis and conjunctivitis (32.1%). Rhinitis was most frequently persistent (90.1%), moderate (80.3%), and partially or poorly controlled (94.3%). Asthma was moderate persistent in almost half of adult (46.3%) and pediatric (42.3%) patients and was partially controlled in over half of adult patients (60.0%) and almost half of pediatric ones (42.3%). The demographic characteristics as well as rhinitis and asthma characteristics across the three age groups are summarized in Table S1.

Most patients were sensitized to other allergens in addition to *O. europaea* and grass pollens according to SPTs (59.6%) and sIgE determinations (86.7%). Additional sensitizations included other aeroallergens such as house dust mites, plane tree pollen, cypress pollen, fungal allergens, and animal epithelia. However, these sensitizations were not considered clinically relevant by the treating allergist and were therefore not regarded as the main drivers of the patients’ seasonal respiratory symptoms or the indication for allergen immunotherapy.

### 3.2. Tolerability of AIT

Seventeen patients (7.8%) experienced a total of 18 adverse reactions (12 in adults and 6 in children), which were all LRs, and mostly immediate (61.1%), and occurred mainly in the up-dosing phase (77.8%) (Table 3). No severe or serious reactions were reported. The 18 ARs occurred in 2528 injections, corresponding to an overall rate of adverse reactions of 0.72 per 100 injections.

The occurrence of adverse reactions lacked any relationships with demographic variables, including age group and sex, and clinical variables, including rhinitis and asthma diagnosis (Table 4).

### 3.3. Changes in Rhinitis Symptoms Before and After AIT

**Figure 1:**
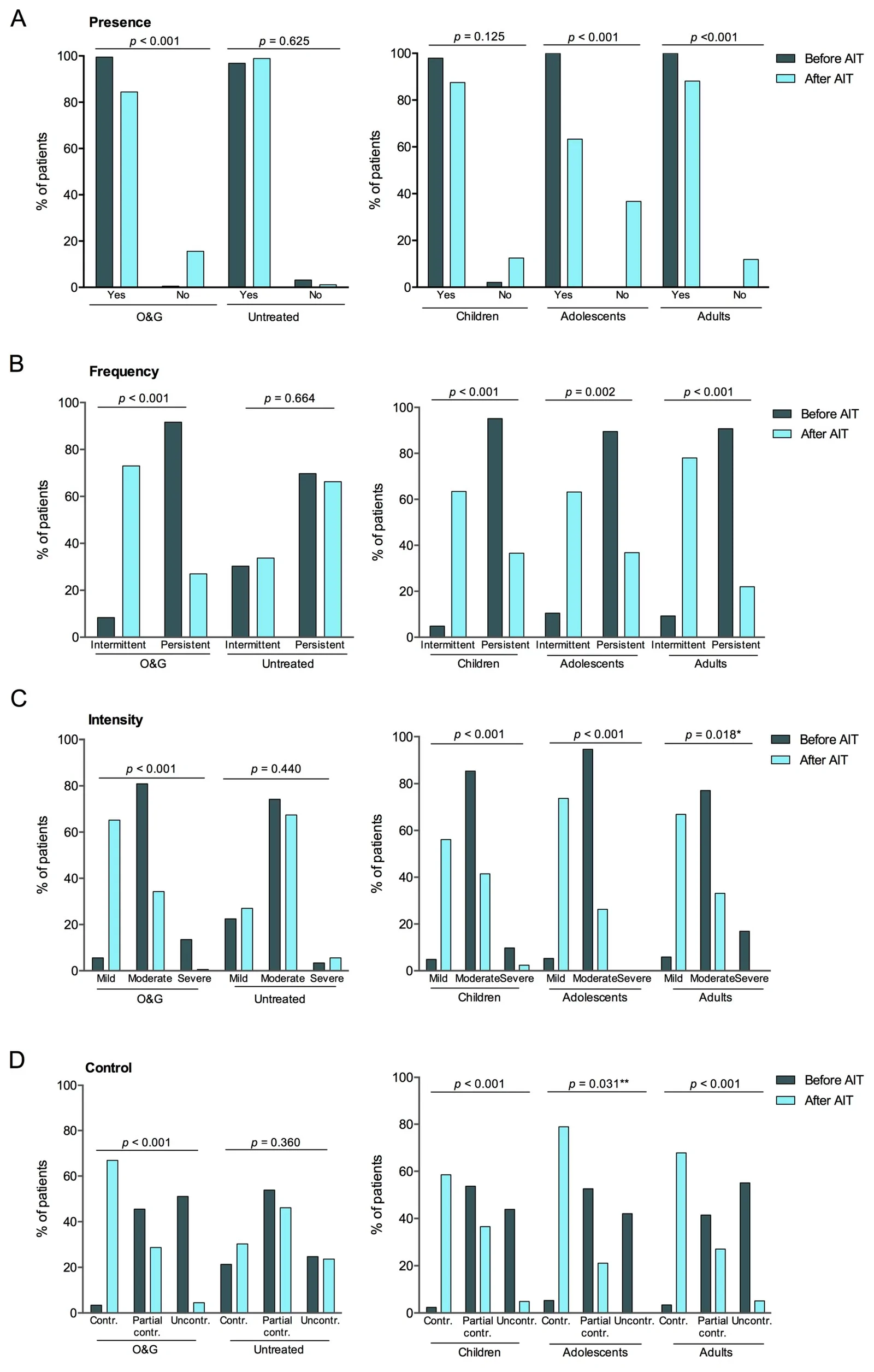
Rhinitis symptoms before and after AIT, including presence (**A**), frequency (**B**), severity (**C**), and control (**D**), based on the Allergic Rhinitis and its Impact on Asthma (ARIA) classification according to treatment (O&G vs. untreated) and, in treated patients, according to age group (children, adolescents, and adults). AIT, allergen immunotherapy. *p*-values comparing the distribution of patients before and after AIT were calculated using the McNemar test except for * *p*-value calculated using the Chi square statistic with a 95% confidence and ** *p*-value calculated using Fisher’s one-sided statistic.

Patients reporting rhinitis symptoms significantly decreased after AIT by −15.1% percent-points in the O&G group (*p* < 0.001), but not in the UT group (*p* = 0.625). Changes were consistent across age groups, with decreases of −10.4 points in children (*p* = 0.125), which were not statistically significant, and significant decreases of −36.7 points in adolescents (*p* < 0.001) and −17.9 points in adults (*p* < 0.001) (Figure 1A). Rhinitis frequency shifted from mostly persistent to intermittent in the O&G group, representing a −64.6% percent-point decrease in persistent (*p* < 0.001) and remained unchanged in the UT group (*p* = 0.664). Changes were consistent and statistically significant across age groups, with decreases of −58.5 points (*p* < 0.001), −52.7 points (*p* = 0.002), and −68.7 points (*p* < 0.001) in children, adolescents, and adults, respectively (Figure 1B). Rhinitis severity shifted towards mild after AIT, with a +59.6 points increase in this category and concomitant decreases in moderate and severe rhinitis of −46.6 points and −12.9 points (*p* < 0.001), respectively, and remained unchanged in the UT group (*p* = 0.440) (Figure 1C). These observations were consistent across age groups, with increased mild rhinitis by +51.2 points in children (*p* < 0.001), +68.4 points in adolescents (*p* < 0.001,) and 61.0% in adults (*p* = 0.018). Rhinitis control also shifted towards more favorable categories overall (+63.5 points increase in controlled rhinitis; *p* < 0.001) and across age groups, including children (+56.1 points, *p* < 0.001), adolescents (+73.6 points, *p* = 0.031), and adults (+64.4 points, *p* < 0.001), but not in the UT group (*p* = 0.360) (Figure 1D).

### 3.4. Changes in Asthma Symptoms Before and After AIT

**Figure 2:**
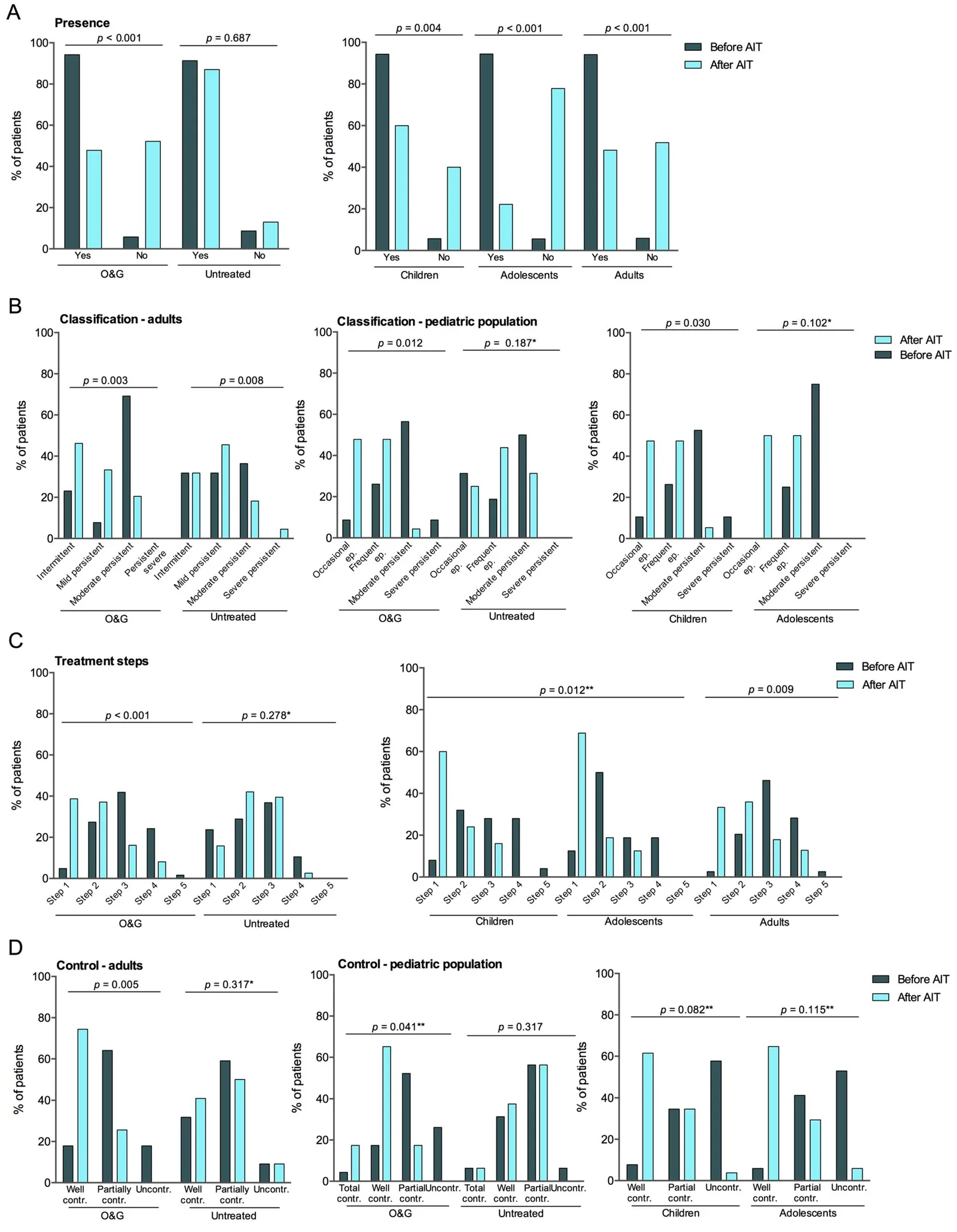
Asthma symptoms before and after AIT, including presence (**A**), classification based on GEMA (**B**), treatment steps (**C**), and control (**D**), according to treatment (O&G vs. untreated) and, in treated patients, age group (children, adolescents, and adults). Asthma classification and control use different categories in adults and pediatric populations, and are represented in different graphs. AIT, allergen immunotherapy. *p*-values comparing the distribution of patients before and after AIT were calculated using the McNemar test except for * *p*-value calculated using the Chi square statistic with a 95% confidence and ** post hoc analysis using the Wilcoxon signed-rank test.

The proportion of patients reporting asthma symptoms before treatment decreased by −46.4% one year after AIT (*p* < remained unchanged in untreated patients (*p* = 0.687). The decrease was consistent across age groups, although more pronounced in adolescents (−72.2%, *p* < 0.0001) compared to adults (−45.9%; *p* < 0.0001) and children (−34.3%; *p* = 0.004) (Figure 2A).

In adults, asthma classification shifted from mostly moderate persistent (69.2%) to intermittent (46.2%) and mild persistent (33.3%), representing a −48.7% decrease in moderate persistent and a +23.1% increase in intermittent in the O&G group (*p* = 0.003). In the UT group, classification significantly shifted (*p* = 0.008), although towards more severe categories, with no change in intermittent and increased moderate persistent (+18.2%) and severe persistent (+4.5%). In the pediatric population in the O&G group, patients with asthma in less severe categories increased by +39.1% and +21.7% corresponding to occasional and frequent episodic, respectively, while moderate persistent decreased (−52.2%) (*p* = 0.012). Changes followed a similar pattern in children and adolescents, with significant −47.3% (*p* = 0.042) and −75.0% (*p* = 0.012) decreases in moderate persistent, respectively (Figure 2B). Categorization according to asthma treatment steps significantly shifted towards lower steps in the O&G group (*p* < 0.001) but not in the UT group (*p* = 0.278). The improvement was consistent across age groups, although changes were only statistically significant in adults (*p* = 0.009) (Figure 2C). Asthma control improved in adults and the pediatric population in the O&G group, with increased well-controlled asthma by +56.5% (*p* = 0.005) in adults and by +65.2% (*p* = 0.041) in the pediatric population, with no significant changes in the corresponding UT groups (*p* = 0.317 for both). Changes followed a similar positive trend in children and adolescents, with increased well-controlled asthma by +47.2% and +50.0%, respectively (Figure 2D).

### 3.5. Changes in the Use of Symptomatic Medication

**Figure 3:**
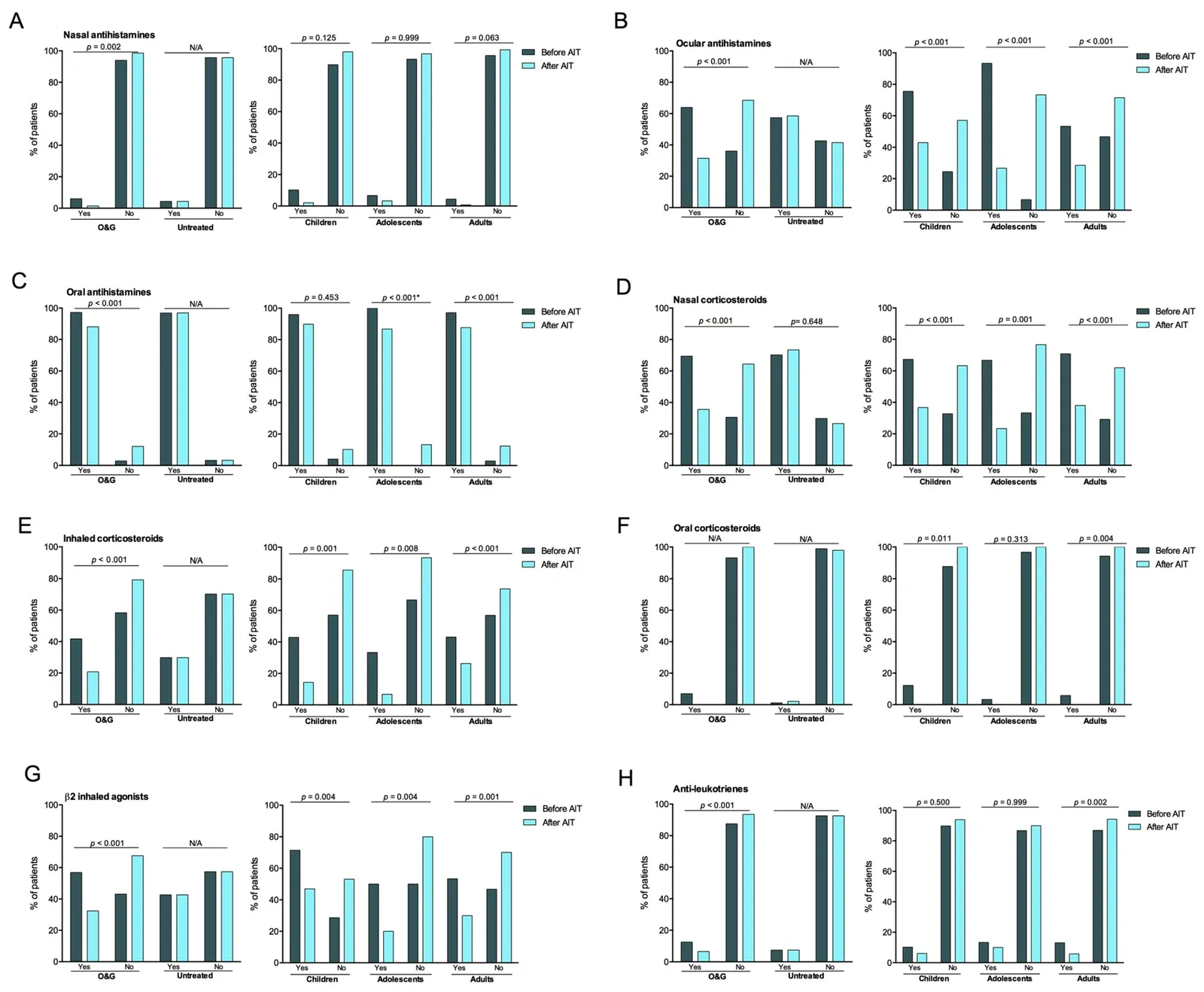
Use of symptomatic medication for rhinitis, conjunctivitis, and asthma before and after AIT, including nasal antihistamines (**A**), ocular antihistamines (**B**), oral antihistamines (**C**), and nasal corticosteroids (**D**), inhaled corticosteroids (**E**), oral corticosteroids (**F**), β2 inhaled agonists (**G**), and anti-leukotrienes (**H**), according to treatment (O&G vs. untreated) and, in treated patients, age group (children, adolescents, and adults). AIT, allergen immunotherapy. *p*-values comparing the distribution of patients before and after AIT were calculated using the McNemar test with a 95% confidence except for * *p*-value calculated using the Chi square statistic with a 95% confidence.

In the O&G group, the use symptomatic medication decreased after treatment, particularly for ocular antihistamines (−32.4-point decrease, *p* < 0.001), nasal corticosteroids (−33.8-point decrease, *p* < 0.001), inhaled corticosteroids (−20.9-point decrease, *p* < 0.001), and β2 inhaled agonists (−24.5-point decrease, *p* < 0.001) and, to a minor extent, for nasal antihistamines (−4.6-point decrease, *p* = 0.002), oral antihistamines (−9.2-point decrease, *p* < 0.001), and anti-leukotrienes (−6.0-point decrease, *p* < 0.001), while no patients used oral corticosteroids after treatment (−6.9-point decrease, *p*, not applicable) (Figure 3). While these trends were consistent across age groups, changes in nasal antihistamines use did not reach statistical significance in the analysis stratified by age, whereas changes in the use of ocular antihistamines, nasal corticosteroids, inhaled corticosteroids, and β2 inhaled agonists were significant in the three age groups. The use of oral antihistamines, oral corticosteroids, andanti-leukotrieness decreased across age groups, reaching statistical significance in specific age groups.

### 3.6. Investigators and Patients’ Perceptions of Treatment Effectiveness

Investigators perceived that most patients in the O&G group had less rhinitis (80.3%), conjunctivitis (80.3%), and asthma symptoms (59.2%) one year after AIT, whereas in the UT groups these percentages were markedly lower (17.0%, 12.8%, and 14.9%, respectively) (*p* < 0.001 across symptoms) (Figure S1). Consistently, investigators perceived that most patients used less symptomatic medication after AIT (86.2%) in the O&G group but not in the UT group (14.9%) (*p* < 0.001). Satisfaction with treatment was rated with a mean (SD) score of 82.0 (14.3) points in the O&G group and 56.24 (21.4) in the UT group (*p* < 0.001).

Patients in the O&G group largely reported less rhinitis, conjunctivitis, and asthma symptoms across age groups, while perceptions in the UT group varied across symptoms and age groups, with same and more symptoms being the most frequent category (Figure S2). Differences in patients’ perceptions between groups were statistically significant across symptoms (*p* < 0.001, except for asthma in children, *p* = 0.002). Accordingly, patients in the O&G largely reported using less medication for rhinitis, conjunctivitis and asthma symptoms one year after AIT, while patients in the UT group reported using the same medication or more across age groups. Differences in patients’ perceptions between groups were statistically significant across medications (*p* < 0.001, except for asthma medication in children, *p* = 0.002, and adolescents *p* = 0.003).

## 4. Discussion

The results from this ambispective, controlled, real-world study evaluating SCIT with undiluted mixtures of *O. europaea* and grass pollen extracts in children, adolescents, and adults showed that this approach resulted in a low incidence of adverse reactions, all of which were local and mild or moderate. Patients in the study cohort were diagnosed with rhinitis and approximately two-thirds had concomitant asthma. Rhinitis and asthma outcomes significantly improved during the pollen season after starting AIT in the O&G group but not in the UT group, including a reduction in the presence of symptoms and a shift towards less severe categories and better control. These improvements were overall consistent across age groups (children, adolescents, and adults) and were accompanied by a decreased use of symptomatic medication. Investigators reported that most patients in the O&G group experienced reduced ARD symptoms during the pollen season after starting SCIT, together with a decrease in medication use and high satisfaction with treatment. Similarly, patients in the O&G group largely reported improvements in rhinitis, conjunctivitis, and asthma symptoms across age groups, with a concomitant decrease in the use of medication.

While SCIT is generally considered safe, SRs including anaphylaxis, although uncommon, may still occur, and remain a main safety concern [25,26]. In our study cohort receiving AIT according to routine practice, adverse reactions were all LRs, were uncommon, and no SRs were reported. Previous studies based on large cohorts or surveys reported SRs rates associated with SCIT ranging from 0.6% to 4.9%, while data from smaller patient series are more variable (3.7–10.6%) [27,28,29,30,31]. Although studies have identified several variables as potentially associated with an increased risk of SRs, uncontrolled asthma has been consistently described as a main risk factor, and guidelines recommend avoiding AIT in these patients [32,33]. Interestingly, in this real-world study, asthma was recorded as uncontrolled or poorly controlled in 12.5% of adults and 19.2% of pediatric patients, who nevertheless received the SCIT without experiencing any SRs. Regarding LRs, the incidence found in this study (7.8%) is in the lower range compared to similarly sized studies assessing SCIT (14.2–34.7%) [30,31]. Overall, our findings support a favorable safety profile in routine clinical practice, both in adult and pediatric patients after a mean of 10.6 months of treatment, despite using undiluted pollen mixtures.

In addition to its favorable safety, the SCIT approach assessed in this study was associated with reductions in rhinitis and asthma symptoms before and after AIT treatment (i.e., after approximately one year of treatment) in routine practice. In contrast, symptoms remained unchanged or worsened in patients who remained on conventional pharmacological treatment (UT group), suggesting a more favorable evolution of ARD in patients receiving AIT. Consistent with these findings, the use of medication to alleviate rhinitis and asthma symptoms decreased across all medications. Moreover, investigators and patients reported a perceived improvement in all symptoms across age groups, along with decreased use of medication. Despite the widespread use of SCIT and the availability of AIT products based on allergoids in Europe, real-world evidence on SCIT with pollen allergoids remains relatively scarce [34]. Nevertheless, our results are consistent with previous reports suggesting that SCIT with single- or multi-allergen pollen and house dust mite allergoids may be effective in reducing rhinitis and asthma symptoms [35,36]. Moreover, previous studies assessing mixtures of *O. europaea* and grass pollen extracts reported similar improvements in allergic symptoms and medication use [37]. Martínez et al. evaluated an undiluted depigmented-polymerized grass/*O. europaea* mixture in 40 pediatric patients and found improvements in rhinoconjunctivitis- and asthma-related quality of life, asthma control, global disease assessment, and rescue medication use with both perennial and pre-co-seasonal schedules [38]. In contrast, the present multicenter study included children, adolescents, and adults and primarily evaluated the tolerability of an undiluted glutaraldehyde-polymerized mixture under real-world clinical practice conditions, with effectiveness assessed through changes in disease classification, symptom control, medication intake, and patient and investigator perceptions. Differences in study population, design, allergen extract formulation, outcome measures, and treatment schedule therefore preclude direct comparisons.

Of note, the formulation used in this study did not contain any adjuvants, including aluminum hydroxide, which has been associated with potential toxicity [39].

Remarkably, most of the variables used to assess disease evolution showed improvement across age groups, particularly in adults and children, while changes in adolescents did not reach statistical significance for specific variables, likely due to the limited representation of this age group. These favorable findings in children are consistent with the previously reported effectiveness of SCIT in this age group [40,41]. Moreover, these findings are particularly relevant considering the reported potential of AIT to modify the disease course, including the prevention of asthma development in children and adolescents [41], although this aspect was not specifically addressed in the present study.

In areas with complex allergen exposure, sensitization to multiple pollens from several species is common and, in Spain, co-sensitization to olive and grass pollen is frequent [8,10,11]. Given the overlapping pollination periods of these species in the month of May, polyallergic patients may experience disease exacerbation and more severe clinical symptoms, particularly during seasons with high pollen counts [42]. In this regard, high olive or grass pollen counts have been associated with asthma epidemics during the pollen season [43,44]. Therefore, in these areas, it is important to target multiple clinically relevant allergens to meet patients’ needs, since single-allergen AIT may be insufficient to adequately control ARD in polyallergic patients [14].

Unlike multiple single-allergen AIT products, the use of allergen mixtures provides a more practical approach. However, their use remains debated, particularly due to concerns regarding extract interactions and effective allergen dosing [16,17,45]. Practical recommendations stress the need to dilute each component in proportion to the number of components in multi-allergen AIT, which may potentially result in allergen dilution to ineffective doses [18]. At the same time, multi-allergen SCIT with undiluted allergen mixtures may raise safety concerns, since it may increase the rate of adverse reactions [17]. Polymerization of allergen extracts with glutaraldehyde decreases their allergenicity by masking IgE-binding epitopes, while maintaining immunogenicity, i.e., the ability to induce T cell-mediated immune tolerance, resulting in increased safety [13]. In this study, the improved safety of glutaraldehyde polymerization may have facilitated the combination of *O. europaea* pollen and grass pollen extracts at maximum concentrations, with an incidence of adverse reactions lower than previously reported [27,28,29,30,31]. Therefore, the novelty of the present study does not lie in demonstrating the general effectiveness of SCIT, which is already well established, but in providing real-world evidence on the tolerability and effectiveness of an undiluted glutaraldehyde-polymerized olive and grass pollen mixture in a relevant sample size population (include a high proportion of pediatrics) of polyallergic patients.

The results from this study should be interpreted considering the general limitations of observational, retrospective studies, including missing data, inherent selection bias, and the nature of the variables used. Standardized variables, such as the combined Symptoms and Medication Score (cSMS), which is the recommended effectiveness measure to reduce heterogeneity among studies, were not assessed. Moreover, the number of SCIT doses received at the time of evaluation (i.e., treatment duration) varied, since study visits matched those scheduled in routine practice and therefore, patients may have received treatment for less than one year. Given that AIT is typically recommended for at least three years to achieve its full disease-modifying effect, the effectiveness findings reported here should be interpreted as early real-world outcomes, as data collection was aligned with the annual follow-up visit routinely performed in clinical practice. Future studies with longer follow-up are warranted to assess the full long-term effectiveness of this AIT approach.

This retrospective study included a comparator group (i.e., control), consisting of patients with AR who did not receive AIT, to reflect the natural evolution of the disease. However, this group was common to all study arms and may not be fully representative of the sensitizing allergens or type of allergy in the O&G group. Therefore, comparisons between groups should be interpreted with caution. Nevertheless, the outcomes of patients in the UT group likely capture the natural evolution of ARD in patients receiving symptomatic treatment, thereby providing contextual support for the observed improvements after initiation of AIT. Despite these limitations, this study offers real-world evidence on the tolerability and effectiveness of SCIT with undiluted *O. europaea*/grass mixtures, including a representation of patients with poly-sensitization and uncontrolled asthma. The findings from this study support a favorable tolerability and effectiveness profile of this SCIT approach for the treatment of allergic rhinitis and asthma. Further prospective studies with longer follow-up and standardized outcome measures are warranted to confirm these findings.

## 5. Conclusions

In this real-world ambispective study, SCIT with undiluted mixtures of *Olea europaea* and grass pollen extracts polymerized with glutaraldehyde was well tolerated with a low incidence of adverse reactions and no SRs reported. This approach was associated with improved allergic rhinitis and asthma symptoms and reduced use of symptomatic medication across age groups. These findings are particularly relevant in settings with a high prevalence of polyallergy, where treatment strategies targeting multiple clinically relevant allergens may be required. Although the results should be interpreted in light of the observational design and study limitations, this study supports the use of undiluted polymerized allergen mixtures as a practical option in routine clinical practice.

## Figures and Tables

**Table 1:** Classification of allergic rhinitis according to ARIA criteria.

Classification	Definition
Intermittent allergic rhinitis	Symptoms occurring on fewer than 4 days per week or for fewer than 4 consecutive weeks.
Persistent allergic rhinitis	Symptoms occurring on 4 or more days per week and for 4 or more consecutive weeks.

**Table 2:** Demographic and clinical characteristics of study patients according to treatment group.

	O&G n = 218	Untreated n = 94
Demographic characteristics		
Sex, male, n (%)	112 (51.4)	47 (50.0)
Age (years), mean (SD)	26.5 (15.6)	25.0 (13.1)
Age groups, n (%)		
Children (5–11 years)	51 (23.4)	14 (14.9)
Adolescents (12–17 years)	30 (13.8)	20 (21.3)
Adults (≥18 years)	137 (62.8)	60 (63.8)
Allergic disease characteristics, n (%)		
Diagnoses		
Rhinitis	215 (98.6)	93 (98.9)
Conjunctivitis	200 (91.7)	70 (74.5)
Asthma	138 (63.3)	44 (46.8)
Diagnoses combinations		
Rhinitis + conjunctivitis + asthma	129 (59.2)	34 (36.2)
Rhinitis + conjunctivitis	70 (32.1)	36 (38.3)
Rhinitis	9 (4.1)	14 (14.9)
Rhinitis + asthma	7 (3.2)	9 (9.6)
Asthma	2 (0.9)	1 (1.1)
Conjunctivitis	1 (0.5)	0 (0)
Rhinitis characteristics (ARIA), n (%)	n = 213	n = 90
Frequency		
Intermittent	21 (9.9)	27 (30.0)
Persistent	192 (90.1)	63 (70.0)
Severity		
Mild	16 (7.5)	20 (22.2)
Moderate	171 (80.3)	66 (73.3)
Severe	26 (12.2)	4 (4.4)
Control		
Controlled	12 (5.6)	19 (21.1)
Partially controlled	101 (47.4)	48 (53.3)
Poor control	100 (46.9)	23 (25.6)
Asthma characteristics (GEMA 5.0), n (%)		
Asthma treatment steps	n = 132	n = 42
Step 1	22 (16.7)	12 (28.6)
Step 2	37 (28.0)	12 (28.6)
Step 3	49 (37.1)	14 (33.3)
Step 4	21 (15.9)	4 (9.5)
Step 5	3 (2.3)	0 (0)
Step 6	0 (0.0)	0 (0)
Classification		
Adults	n = 80	n = 25
Intermittent	25 (21.3)	8 (32.0)
Persistent Mild	17 (21.3)	9 (36.0)
Moderate Persistent	37 (46.3)	8 (32.0)
Severe Persistent	1 (1.3)	0 (0)
Pediatric patients	n = 52	n = 17
Occasional Episodic	10 (19.2)	6 (35.3)
Frequent Episodic	18 (34.6)	3 (17.6)
Moderate Persistent	22 (42.3)	8 (47.1)
Severe Persistent	2 (3.8)	0 (0)
Control		
Adults	n = 80	n = 25
Well-controlled	22 (27.5)	10 (40.0)
Partly controlled	48 (60.0)	13 (52.0)
Uncontrolled	10 (12.5)	2 (8.0)
Pediatric patients	n = 52	n = 17
Complete	1 (1.9)	1 (5.9)
Good	19 (36.5)	6 (35.3)
Partial	22 (42.3)	9 (52.9)
Uncontrolled	10 (19.2)	1 (5.9)

ARIA, Allergic Rhinitis and its Impact on Asthma; GEMA, Spanish Asthma Management Guidelines (Guía Española para el Manejo del Asma); SD, standard deviation. Step 5 corresponds to severe asthma requiring high-dose inhaled corticosteroids plus additional controller therapy. Percentages are calculated based on available data; missing data were not imputed. A small proportion of patients, 3 in the O&G group and 1 in the UT group, presented asthma and/or conjunctivitis only at baseline, reflecting the real-world nature of the study population. As this was an intention-to-treat analysis, these patients were retained to preserve the original study population and minimize potential selection bias.

**Table 3:** Characteristics of adverse reactions to 2528 AIT injections, n = 218.

	Reactions n = 18
Type of reactions	
Local	18 (100)
Systemic	0 (0)
Phase	
Up-dosing	14 (77.8)
Maintenance	4 (22.2)
Onset	
Immediate	11 (61.1)
Late	7 (38.9)
Intensity	
Mild	9 (50.0)
Moderate	9 (50.0)
Severe	0 (0)
Local reaction symptoms	
Erythema	17 (94.6)
Papule formation	16 (88.9)
Pruritus	12 (66.7)
Pain	3 (16.7)
Treatments ^a^	
Local ice	18 (100)
Oral antihistamine	15 (83.3)
Topical corticosteroid	6 (33.3)
Oral corticosteroid	2 (11.1)
Antihistamine IV	0 (0)
Corticosteroids IV/IM	0 (0)

AIT, allergen immunotherapy; IM, intramuscular; IV, intravenous. ^a^ Including intravenous antihistamine, oral corticosteroids, and fluid therapy, among others.

**Table 4:** Relationship between adverse reactions and sociodemographic, clinical, and treatment variables, n (%).

	Patients with ARs n = 17	Patients with No ARs n = 201	Total n = 218	*p*-Value ^a^
Demographic variables				
Age				0.869
Children	6 (54.5)	45 (22.4)	51 (23.4)	
Adolescents	0 (0)	30 (14.9)	30 (13.8)	
Adults	11 (64.7)	126 (62.7)	137 (62.8)	
Sex				0.893
Male	9 (4.1)	103 (47.2)	112 (51.4)	
Female	8 (3.7)	98 (45.0)	106 (48.6)	
Clinical variables				
Rhinitis diagnosis				0.612
Yes	17 (7.8)	198 (90.8)	215 (98.6)	
No	0 (0)	3 (1.4)	3 (1.4)	
Asthma diagnosis				0.516
Yes	12 (5.5)	126 (57.8)	138 (63.3)	
No	5 (2.3)	75 (34.4)	80 (36.7)	

AR, adverse reactions. ^a^ Chi square test with a 95% confidence level.

## Data Availability

The datasets generated and/or analyzed during the current study are available from the corresponding author on reasonable request.

## Supplementary Materials

The following supporting information can be downloaded at https://www.mdpi.com/article/10.3390/diseases14090321/s1. Figure S1: Investigators’ perceptions on changes in rhinitis, conjunctivitis, and asthma symptoms (A) and changes in use of medication (B), and satisfaction with treatment (C), according to treatment (O&G vs. untreated); Figure S2: Patients’ opinion regarding the change in rhinitis, conjunctivitis, and asthma symptoms (A) and use of medication (B), according to treatment (O&G vs. untreated) and age (children, adolescents, and adults). Table S1: Demographic and clinical characteristics of study patients in the O&G group overall and according to age groups; Table S2: Patients’ sensitization profile based on skin prick test and specific IgE determinations (O&G group).

## Abbreviations

The following abbreviations are used in this manuscript:

AIT	Allergen immunotherapy
AR	Adverse reaction
ARD	Allergic respiratory disease
ARIA	Allergic Rhinitis and its Impact on Asthma
cSMS	Combined Symptoms and Medication Score
GEMA	Guía Española para el manejo del asma
GINA	Global Initiative for Asthma
LR	Local reaction
O&G	Olive and grass pollen allergen immunotherapy treatment
SCIT	Subcutaneous immunotherapy
sIgE	Specific IgE
SLIT	Sublingual immunotherapy
SPT	Skin prick test
SR	Systemic reaction
UT	Untreated
VAS	Visual analog scale
WAO	World Allergy Organization

## References

[1] Bousquet J., Anto J.M., Bachert C., Baiardini I., Bosnic-Anticevich S., Walter Canonica G., Melén E., Palomares O., Scadding G.K., Togias A. (2020). Allergic Rhinitis. Nat. Rev. Dis. Primers.

[2] Papi A., Brightling C., Pedersen S.E., Reddel H.K. (2018). Asthma. Lancet.

[3] Licari A., Magri P., Silvestri A.D., Giannetti A., Indolfi C., Mori F., Marseglia G.L., Peroni D. (2023). Epidemiology of Allergic Rhinitis in Children: A Systematic Review and Meta-Analysis. J. Allergy Clin. Immunol. Pract..

[4] Savouré M., Bousquet J., Jaakkola J.J.K., Jaakkola M.S., Jacquemin B., Nadif R. (2022). Worldwide Prevalence of Rhinitis in Adults: A Review of Definitions and Temporal Evolution. Clin. Transl. Allergy.

[5] Song P., Adeloye D., Salim H., Dos Santos J.P., Campbell H., Sheikh A., Rudan I. (2022). Global, regional, and national prevalence of asthma in 2019: A systematic analysis and modelling study. J. Glob. Health.

[6] Dierick B.J.H., van der Molen T., Flokstra-de Blok B.M.J., Muraro A., Postma M.J., Kocks J.W.H., van Boven J.F.M. (2020). Burden and Socioeconomics of Asthma, Allergic Rhinitis, Atopic Dermatitis and Food Allergy. Expert Rev. Pharmacoecon. Outcomes Res..

[7] Linneberg A., Dam Petersen K., Hahn-Pedersen J., Hammerby E., Serup-Hansen N., Boxall N. (2016). Burden of Allergic Respiratory Disease: A Systematic Review. Clin. Mol. Allergy.

[8] Dramburg S., Grittner U., Potapova E., Travaglini A., Tripodi S., Arasi S., Pelosi S., Acar Şahin A., Aggelidis X., Barbalace A. (2024). Heterogeneity of Sensitization Profiles and Clinical Phenotypes among Patients with Seasonal Allergic Rhinitis in Southern European Countries—The @IT.2020 Multicenter Study. Allergy.

[9] Cosme J., Pedro E., Pereira-Santos M.C., Anabela A., Caiado J., Paulino M., Tomás E., Farinha S., Matos E., Pinela A. (2024). Molecular Sensitization Profile to Grass and Olive Pollens in Portugal. Eur. Ann. Allergy Clin. Immunol..

[10] Moreno C., Justicia J.L., Quiralte J., Moreno-Ancillo Á., Iglesias-Cadarso A., Torrecillas M., Labarta N., García M.A., Dávila I. (2014). Olive, Grass or Both? Molecular Diagnosis for the Allergen Immunotherapy Selection in Polysensitized Pollinic Patients. Allergy.

[11] Martínez-Cañavate Burgos A., Torres-Borrego J., Molina Terán A.B., Corzo J.L., García B.E., Rodríguez Pacheco R., Moreno Aguilar C., Dávila I. (2018). Molecular Sensitization Patterns and Influence of Molecular Diagnosis in Immunotherapy Prescription in Children Sensitized to Both Grass and Olive Pollen. Pediatr. Allergy Immunol..

[12] Durham S.R., Shamji M.H. (2023). Allergen Immunotherapy: Past, Present and Future. Nat. Rev. Immunol..

[13] Compalati E., Incorvaia C., Cavaliere C., Masieri S., Gargiulo A., Mistrello G., Frati F. (2020). The Role of Allergoids in Allergen Immunotherapy: From Injective to Sublingual Route. Eur. Ann. Allergy Clin. Immunol..

[14] Viñas M., Bellido V., El-Qutob D., García F., Jimenez-Rodriguez T.W., Rial M., Vega-Chicote J.M., Cuesta-Herranz J. (2026). The Spanish REAL Project: Expert Allergist Guidance on Allergen Immunotherapy Based on Mixtures from Different Allergenic Sources. Front. Allergy.

[15] Nelson H.S. (2021). How Important Is Proper Dosing for Subcutaneous and Sublingual Allergy Immunotherapy?. Allergy Asthma Proc..

[16] Incorvaia C., Frati F., Puccinelli P., Riario-Sforza G.G., Bo S.D. (2006). Dose Dependence of Efficacy and Safety of Subcutaneous Immunotherapy. Monaldi Arch. Chest Dis..

[17] Nony E., Martelet A., Jain K., Moingeon P. (2016). Allergen Extracts for Immunotherapy: To Mix or Not to Mix?. Expert Rev. Clin. Pharmacol..

[18] Demoly P., Passalacqua G., Pfaar O., Sastre J., Wahn U. (2016). Management of the Polyallergic Patient with Allergy Immunotherapy: A Practice-Based Approach. Allergy Asthma Clin. Immunol..

[19] Verdeguer Segarra O., Almeida Sánchez Z., Quarta S., Funes Vera E., González Jiménez Ó.M., Hernández Santana G., Herrero Lifona L., López-González P., Martínez-Gomariz M., López-Cauce B. (2026). Safety and Effectiveness of Subcutaneous Immunotherapy with a Glutaraldehyde-Polymerized Mite Allergen Extract in Adults and Children with Allergic Rhinitis with or Without Asthma Due to Dermatophagoides. Diseases.

[20] Von Elm E., Altman D.G., Egger M., Pocock S.J., Gøtzsche P.C., Vandenbroucke J.P., STROBE Initiative (2008). The Strengthening the Reporting of Observational Studies in Epidemiology (STROBE) Statement: Guidelines for Reporting Observational Studies. J. Clin. Epidemiol..

[21] Cox L., Larenas-Linnemann D., Lockey R.F., Passalacqua G. (2010). Speaking the Same Language: The World Allergy Organization Subcutaneous Immunotherapy Systemic Reaction Grading System. J. Allergy Clin. Immunol..

[22] Brożek J.L., Bousquet J., Agache I., Agarwal A., Bachert C., Bosnic-Anticevich S., Brignardello-Petersen R., Canonica G.W., Casale T., Chavannes N.H. (2017). Allergic Rhinitis and Its Impact on Asthma (ARIA) Guidelines—2016 Revision. J. Allergy Clin. Immunol..

[23] Comité Ejecutivo GEMA 5.0 GEMA 5.0—Guía Para El Manejo Del Asma. https://www.semg.es/index.php/consensos-guias-y-protocolos/327-gema-5-0-guia-espanola-para-el-manejo-del-asma.

[24] Gaig P., Ferrer M., Muñoz-Lejarazu D., Lleonart R.R., García-Abujeta J.L., Caballero T., Rodríguez A., Echechipia S., Martínez-Cocera C., Domínguez F.J. (2004). Prevalencia de Alergia En La Población Adulta Española. Alergol. Inmunol. Clín..

[25] Incorvaia C., Cavaliere C., Schroeder J.W., Leo G., Nicoletta F., Barone A., Ridolo E. (2023). Safety and Adverse Reactions in Subcutaneous Allergen Immunotherapy: A Review. Acta Biomed. Atenei Parm..

[26] Incorvaia C., Pucciarini F., Makri E., Gritti B.L., Ridolo E. (2020). Allergen Immunotherapy for Respiratory Allergy: To What Extent Can the Risk of Systemic Reactions Be Reduced?. Expert Opin. Drug Saf..

[27] Bona D.D., Magistà S., Masciopinto L., Lovecchio A., Loiodice R., Bilancia M., Albanesi M., Caiaffa M.F., Nettis E., Macchia L. (2020). Safety and Treatment Compliance of Subcutaneous Immunotherapy: A 30-Year Retrospective Study. Respir. Med..

[28] Epstein T.G., Liss G.M., Berendts K.M., Bernstein D.I. (2019). AAAAI/ACAAI Subcutaneous Immunotherapy Surveillance Study (2013–2017): Fatalities, Infections, Delayed Reactions, and Use of Epinephrine Autoinjectors. J. Allergy Clin. Immunol. Pract..

[29] Calderón M.A., Vidal C., Rodríguez Del Río P., Just J., Pfaar O., Tabar A.I., Sánchez-Machín I., Bubel P., Borja J., Eberle P. (2017). European Survey on Adverse Systemic Reactions in Allergen Immunotherapy (EASSI): A Real-Life Clinical Assessment. Allergy.

[30] Zhang W., Deng Y., Tong H., Xiang R., Chen S., Kong Y., Tao Z., Xu Y. (2021). Adverse Reactions to Subcutaneous Immunotherapy in Patients with Allergic Rhinitis, a Real-World Study. Eur. Arch. Oto-Rhino-Laryngol..

[31] Coutinho C., Lourenco T., Fernandes M., Lopes A., Spinola Santos A., Pereira Barbosa M. (2022). Subcutaneous Immunotherapy with Aeroallergens: Safety Profile Assessment. Eur. Ann. Allergy Clin. Immunol..

[32] Nelson H.S. (2020). Allergy Immunotherapy for Inhalant Allergens: Strategies to Minimize Adverse Reactions. Allergy Asthma Proc..

[33] Pitsios C., Demoly P., Bilò M.B., Gerth van Wijk R., Pfaar O., Sturm G.J., Rodriguez del Rio P., Tsoumani M., Gawlik R., Paraskevopoulos G. (2015). Clinical Contraindications to Allergen Immunotherapy: An EAACI Position Paper. Allergy.

[34] Mahler V., Esch R.E., Kleine-Tebbe J., Lavery W.J., Plunkett G., Vieths S., Bernstein D.I. (2019). Understanding Differences in Allergen Immunotherapy Products and Practices in North America and Europe. J. Allergy Clin. Immunol..

[35] Nevot-Falcó S., Mancebo E.G., Martorell A., Calatayud C.M., Perotti S.C., Rojas M.J., Rodriguez-Rodriguez M., Padial M.A., El Qutob Lopez F.D., Perez-Camo I. (2021). Safety and Effectiveness of a Single Multiallergen Subcutaneous Immunotherapy in Polyallergic Patients. Int. Arch. Allergy Immunol..

[36] Santaolalla M., Arias-Irigoyen J., Soler J.M., Duque J.M., Escudero R., Pérez-Formoso J.L., Lobera T., Rueda M., Alias C., Hermida H. (2024). Efficacy and Safety of Subcutaneous Immunotherapy with Polymerized Allergen Mixtures in Polyallergic Patients—ARES Observational Study. Expert Rev. Clin. Immunol..

[37] Pérez Montero A., Sanz-Rosa D., Carnés J. (2024). Retrospective, Real Life Study on the Effectiveness and Safety of a Depigmented-Polymerized Subcutaneous Vaccine Containing a Mixture of Grasses and Olea Europaea. Int. Arch. Allergy Immunol..

[38] Martinez A., Torres J., Molina A.B., Muños C., Diaz M., Corzo J.L., Echeverria L., Sanchez J., Ruiz M. (2021). Evaluation of a Pre-Co-Seasonal and a Perennial Schedule of a Single Multiallergen Depigmented-Polymerized Subcutaneous Immunotherapy in Paediatric Patients. Allergol. Immunopathol..

[39] Jensen-Jarolim E., Bachmann M.F., Bonini S., Jacobsen L., Jutel M., Klimek L., Mahler V., Mösges R., Moingeon P., O’Hehir R.E. (2020). State-of-the-Art in Marketed Adjuvants and Formulations in Allergen Immunotherapy: A Position Paper of the European Academy of Allergy and Clinical Immunology (EAACI). Allergy.

[40] Larenas-Linnemann D.E.S., Pietropaolo-Cienfuegos D.R., Calderón M.A. (2011). Evidence of Effect of Subcutaneous Immunotherapy in Children: Complete and Updated Review from 2006 Onward. Ann. Allergy Asthma Immunol..

[41] Halken S., Larenas-Linnemann D., Roberts G., Calderón M.A., Angier E., Pfaar O., Ryan D., Agache I., Ansotegui I.J., Arasi S. (2017). EAACI Guidelines on Allergen Immunotherapy: Prevention of Allergy. Pediatr. Allergy Immunol..

[42] Florido J.F., Delgado P.G., De San Pedro B.S., Quiralte J., De Saavedra J.M.A., Peralta V., Valenzuela L.R. (1999). High Levels of Olea Europaea Pollen and Relation with Clinical Findings. Int. Arch. Allergy Immunol..

[43] Porcel Carreño S., Gómez Nieves E., Fernández-Caldas E., Abel Fernández E., Cases B., Tudela J., Maghfour Martin Y., Domínguez Domínguez E., Alvarado Arenas M., Jiménez Timón S. (2020). Immunochemical and Physical Quantitation of Grass and Olive Pollen Allergens: Correlation With Asthma Admissions in Cáceres, Spain. J. Investig. Allergol. Clin. Immunol..

[44] Galán I., Prieto A., Rubio M., Herrero T., Cervigón P., Cantero J.L., Gurbindo M.D., Martínez M.I., Tobías A. (2010). Association between Airborne Pollen and Epidemic Asthma in Madrid, Spain: A Case–Control Study. Thorax.

[45] Nelson H.S. (2021). To Mix or Not to Mix in Allergy Immunotherapy Vaccines. Curr. Opin. Allergy Clin. Immunol..

