# Supplementary material for: Safety and Effectiveness of Subcutaneous Immunotherapy with an Undiluted Glutaraldehyde-Polymerized Pollen Extract Mixture in Adults and Children with Allergic Rhinitis with or Without Asthma Due to Olive and Grass Pollen
Source: Diseases. 2026 Sep 4;14(9):321. doi: 10.3390/diseases14090321 (PMC13605683; doi:10.3390/diseases14090321)
Supplement: Supplementary file 1 [file diseases-14-00321-s001.zip › diseases-4460955-supplementary.pdf]

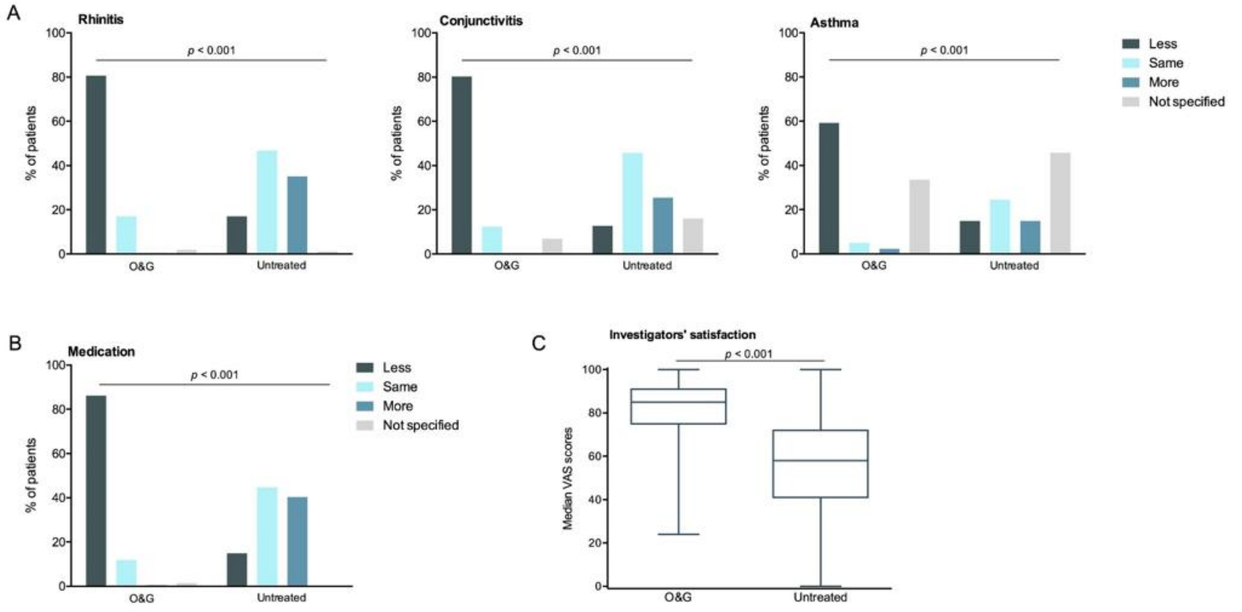

Figure S1. Investigators' perceptions on changes in rhinitis, conjunctivitis, and asthma symptoms (A) and changes in use of medication (B), and satisfaction with treatment (C), according to treatment (O&G vs. untreated). AIT, allergen immunotherapy. In (C), the box represents the interquartile range (IQR: Q1, Q3), the line in the middle of the box represents the median, and the whiskers represent the minimum and maximum. *P*-values were calculated using the Chi-square test (A and B) and the Mann Whitney test (C) with 95% confidence.

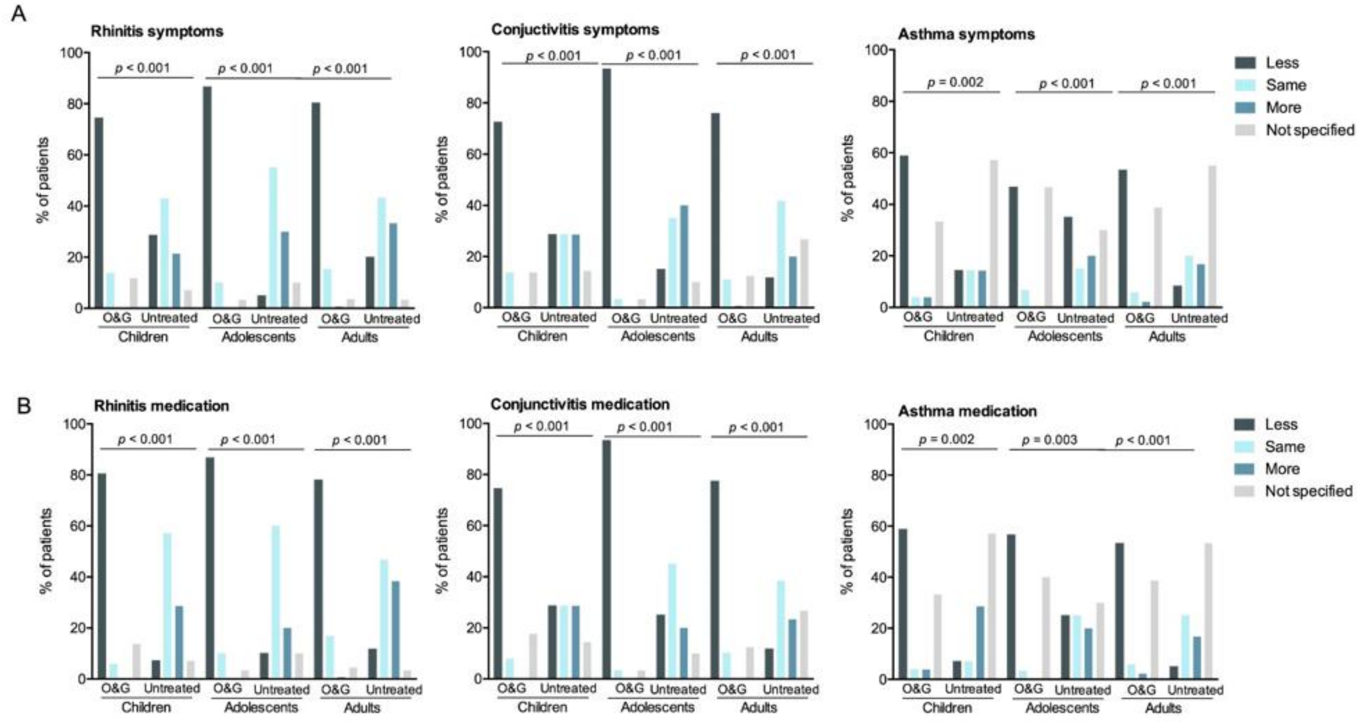

Figure S2. Patients' opinion regarding the change in rhinitis, conjunctivitis, and asthma symptoms (A) and use of medication (B), according to treatment (O&G vs untreated) and age (children, adolescents, and adults). AIT, allergen immunotherapy. *P*-values were calculated using the Chi-square test with 95% confidence.

Table S1. Demographic and clinical characteristics of study patients in the O&G group overall and according to age groups.

	Age Groups			Total n=218
	**Children n=51**	**Adolescents n=30**	**Adults n=137**	
**Demographic characteristics**				
Sex, *n (%)*				
Male	35 (68.6)	16 (53.3)	61 (44.5)	112 (51.4)
Female	16 (31.4)	14 (46.7)	76 (55.5)	106 (48.6)
Age (years), *mean (SD)*	9.1 (1.7)	14.4 (1.8)	35.7 (12.6)	26.5 (15.6)
**Allergic disease characteristics**				
Diagnoses, *n (%)*				
Rhinitis	50 (98.0)	30 (100.0)	135 (98.5)	215 (98.6)
Conjunctivitis	50 (98.0)	30 (100.0)	120 (87.6)	200 (91.7)
Asthma	37 (72.5)	17 (56.7)	84 (61.3)	138 (63.3)
**Rhinitis characteristics (ARIA), *n (%)***	n=49	n=30	n=134	n=213
Frequency				
Intermittent	2 (4.1)	4 (13.3)	15 (11.2)	21 (9.9)
Persistent	47 (95.9)	26 (86.7)	119 (88.8)	192 (90.1)
Severity				
Mild	2 (4.1)	5 (16.7)	9 (6.7)	16 (7.5)
Moderate	43 (87.8)	25 (83.3)	103 (76.9)	171 (80.3)
Severe	4 (8.2)	0 (0.0)	22 (16.4)	26 (12.2)
Control				
Controlled	2 (4.1)	2 (6.7)	8 (6.0)	12 (5.6)
Partially controlled	27 (55.1)	17 (56.7)	57 (42.5)	101 (47.4)
Poor control	20 (40.8)	11 (36.7)	69 (51.5)	100 (46.9)
**Asthma characteristics (GEMA 5.0), *n (%)***	n=35	n=17	n=80	n=132
Asthma treatment steps				
Step 1	6 (17.1)	2 (11.8)	14 (17.5)	22 (16.7)
Step 2	10 (28.6)	8 (47.1)	19 (23.8)	37 (28.0)
Step 3	14 (40.0)	4 (23.5)	31 (38.8)	49 (37.1)
Step 4	4 (11.4)	3 (17.6)	14 (17.5)	21 (15.9)
Step 5	1 (2.9)	0 (0.0)	2 (2.5)	3 (2.3)
Step 6	0 (0.0)	0 (0.0)	0 (0.0)	0 (0.0)
Not available	0 (0.0)	1 (14.3)	1 (2.2)	2 (3.0)
Classification				
Adults, n=80				
Intermittent	N/A	N/A	25 (31.3)	N/A
Mild Persistent	N/A	N/A	17 (21.3)	N/A
Moderate Persistent	N/A	N/A	37 (46.3)	N/A
Severe Persistent	N/A	N/A	1 (1.3)	N/A
Pediatric patients, n=52				
Occasional Episodic	7 (20.0)	3 (17.6)	N/A	N/A
Frequent Episodic	11 (31.4)	7 (41.2)	N/A	N/A
Moderate Persistent	15 (42.9)	7 (41.2)	N/A	N/A
Severe Persistent	2 (5.7)	0 (0.0)	N/A	N/A
Control				
Adults, n=80				
Well controlled	N/A	N/A	22 (27.5)	N/A
Partially controlled	N/A	N/A	48 (60.0)	N/A
Bad controlled	N/A	N/A	10 (12.5)	N/A
Pediatric patients, n=52				
Total	1 (2.9)	0 (0.0)	N/A	N/A
Good	11 (31.4)	8 (47.1)	N/A	N/A

Partial	14 (40.0)	8 (47.1)	N/A	N/A
Poor	9 (25.7)	1 (5.9)	N/A	N/A

ARIA, Allergic Rhinitis and its Impact on Asthma; GEMA, Spanish Asthma. Management Guidelines (Guía Española para el Manejo del Asma); N/A, not applicable.

Table S2. Patients' sensitization profile based on skin prick test and specific IgE determinations (O&G group).

**Skin prick test**	
Number of sensitizations, *mean (SD)/range* n=206	3.05 (2.34)/0–11
Sensitizations, *n (%)* n=218	
O. europaea + grass + other allergens	92 (42.2)
O. europaea + other allergens	36 (16.5)
O. europaea + grass	22 (10.1)
Grass + other allergens	2 (0.9)
Not available	66 (30.3)
**Specific IgE**	
Number of sensitizations, *mean (SD)/range* n=190	5.0 (2.05)/1–11
Sensitizations, *n (%)* n=218	
O. europaea + other allergens	165 (75.7)
O. europaea + Grass + other allergens	22 (10.1)
Other allergens	2 (0.9)
O. europaea + Grass	1 (0.5)
Not available	28 (12.8)

SD, standard deviation. sIgE, specific immunoglobulin E. In addition to olive and grass pollens, the allergen sources individually recorded during routine clinical assessment included *Cupressus arizonica, Dermatophagoides pteronyssinus, D. farinae, Blomia tropicalis, Lepidoglyphus destructor, Platanus acerifolia, Quercus ilex, Alternaria alternata*, and cat and dog epithelia, as well as other aeroallergens and allergen components. The allergen sources assessed varied among patients according to routine clinical practice at the participating centers. Additional sensitizations were not considered clinically relevant for allergen immunotherapy prescription according to the treating allergist's clinical judgment.
